# Celiac disease

**DOI:** 10.1186/1750-1172-1-3

**Published:** 2006-03-01

**Authors:** Wolfgang Holtmeier, Wolfgang F Caspary

**Affiliations:** 1 Medizinische Klinik I, Johann Wolfgang Goethe-Universität, Theodor-Stern-Kai 7, 60590 Frankfurt am Main, Germany

## Abstract

Celiac disease is a chronic intestinal disease caused by intolerance to gluten. It is characterized by immune-mediated enteropathy, associated with maldigestion and malabsorption of most nutrients and vitamins. In predisposed individuals, the ingestion of gluten-containing food such as wheat and rye induces a flat jejunal mucosa with infiltration of lymphocytes. The main symptoms are: stomach pain, gas, and bloating, diarrhea, weight loss, anemia, edema, bone or joint pain. Prevalence for clinically overt celiac disease varies from 1:270 in Finland to 1:5000 in North America. Since celiac disease can be asymptomatic, most subjects are not diagnosed or they can present with atypical symptoms. Furthermore, severe inflammation of the small bowel can be present without any gastrointestinal symptoms. The diagnosis should be made early since celiac disease causes growth retardation in untreated children and atypical symptoms like infertility or neurological symptoms. Diagnosis requires endoscopy with jejunal biopsy. In addition, tissue-transglutaminase antibodies are important to confirm the diagnosis since there are other diseases which can mimic celiac disease. The exact cause of celiac disease is unknown but is thought to be primarily immune mediated (tissue-transglutaminase autoantigen); often the disease is inherited. Management consists in life long withdrawal of dietary gluten, which leads to significant clinical and histological improvement. However, complete normalization of histology can take years.

## Disease name and synonyms

Celiac disease (CD) in children and celiac sprue in adults are probably the same disorder with the same pathogenesis. The synonyms are: Coeliac disease (British spelling) – Celiac sprue – Nontropical sprue-Gluten-sensitive enteropathy – Idiopathic steatorrhea

## Definition

Celiac disease is a chronic intestinal disease mostly associated with malabsorption caused by intolerance to gluten. It is characterized by immune-mediated enteropathy (villous flattening), resulting in maldigestion and malabsorption. Clinical and histological improvement can be obtained after withdrawal of dietary gluten.

## Differential diagnosis

Celiac disease is characterized by malabsorption and villous atrophy. However, diseases other than CD can cause marked villous flattening and increased intraepithelial lymphocytes (IEL) [1]. Differential diagnosis is of special importance for subjects in whom CD is suspected and who have negative serology. The following diseases, which can have similar features, must be ruled out [1-4]:

• Tropical sprue

• Collagenous colitis

• Whipple's disease

• Giardiasis

• Viral enteritis

• AIDS

• Crohn's disease of the small intestine

• Small intestinal lymphoma

• Carbohydrate intolerance, cow's milk intolerance

• Autoimmune enteropathy

• Graft-*vs*-host disease

• Radiation damage

## Epidemiology

Prevalence of clinically overt celiac disease varies from 1/270 in Finland to 1/5,000 in North America. However, since celiac disease can be asymptomatic, most subjects are not diagnosed or they can present with atypical symptoms. In epidemiological studies aimed to assess CD prevalence, large cohorts in North America and Europe were screened for highly-sensitive endomysium or tissue transglutaminase antibodies. Besides, they underwent subsequent small intestinal biopsies when antibody testing was positive. The CD prevalence was found to be much higher than expected. Approximately 1/100 to 1/500 were found positive for antibodies and had villous atrophy of the small intestine [5-10]. Thus, up to 1% of a western population tests positive for celiac disease. There are approximately 7–10 undiagnosed subjects for each known CD patient. Furthermore, approximately 10% of the first-degree relatives also have CD [11,12].

## Clinical description

Celiac disease is diagnosed typically in early childhood around age of 2 years. A second peak is found around age of 40 years [3]. Most symptoms are due to malabsorption of nutrients and vitamins [13,14]. However, the clinical manifestations differ greatly, depending on each case and ranging from ***asymptomatic (silent) ***[15] to ***full blown (symptomatic, clinically overt) celiac disease ***[16]. The severity of symptoms is not necessarily proportional to the severity of the mucosal lesions and patients with total villous atrophy can be asymptomatic or present with subclinical symptoms such as iron deficiency or muscle cramps. Nowadays, more subjects present with asymptomatic or mild celiac disease than with the classical symptoms of severe malabsorption [4,17].

The term ***"atypical" celiac disease ***is used for patients who present with extraintestinal symptoms like Immunoglobulin A (IgA)-nephropathy, hemosiderosis of the lungs and a variety of neurological diseases. Antibodies and typical small intestinal changes can be found. Early diagnosis is desirable since many of these symptoms can disappear after the initiation of a gluten-free diet.

The term ***"latent" celiac disease ***refers to subjects who will develop the disease later in life but who do not have a flat mucosa despite a gluten-containing diet [17-20]. Increased intraepithelial lymphocytes (IEL) and positive endomysium antibody (EMA) or positive tissue transglutaminase (tTG) antibody tests are sometimes found in these subjects [21-23]. What triggers the onset of the disease in these subjects remains unknown.

Ferguson *et al*. introduced the term "potential" celiac disease in 1993 to characterize in details patients with latent CD [24]. The authors suggested "potential" CD to be used for the subjects who have markers of latent CD (elevated IEL, positive for tTG) without ever developing overt CD, with "latent" CD being used for patients who will develop a flat mucosa in the future. However, this discrimination is very artificial and not shared by other specialists in the field [25]. The terms "latent" and "potential" celiac disease are not used by all authors in the same way, which can further confuse matters. Patients with latent or potential celiac disease may develop symptoms that respond to a gluten-free diet [26]. For the definition of the different states of celiac disease, see table 1.

**Table 1:** Definition of different states of celiac disease.

**States of celiac disease (CD)**	**Definition**
Clinically overt CD	Typical gastrointestinal symptoms and signs of malabsorption. Histological changes with villous atrophy and hypertrophic crypts (Marsh type-3 lesion, see table 2).
Symptomatic (active) CD	Same findings as in clinically overt CD
Silent CD	Asymptomatic patients with typical histological changes (Marsh type-3)
Asymptomatic CD	Same findings as in silent CD
Atypical CD	Extraintestinal findings such as IgA-nephropathy and neurological symptoms. Typical histological changes.
Latent CD/potential CD	Subjects with genetic predisposition who have initially a normal histology with no atrophy or crypt hyperplasia. Immunological abnormalities such as increased count of IELs (particularly gamma-delta T cells, Marsh type-1) and positive EMA or tTG-antibody tests are sometimes present. These subjects may develop clinically overt CD later in life.
Refractory CD	Patients who do not respond to a gluten-free diet or who previously responded but later become non-responsive to a gluten-free diet. Intestinal lymphoma may have developed. Inadvertent gluten ingestion and other diseases must be excluded (see differential diagnosis).

Celiac disease is also associated with several extraintestinal diseases and autoimmune diseases, which can not be linked to nutrient deficiencies [27-33]. For example, up to 8% of patients with type 1 diabetes were reported to test positive for CD [4]. CD patients are also at higher risk of developing malignancies. Holmes *et al*. reported an increased risk especially of intestinal lymphoma in subjects with untreated celiac disease compared to patients on a gluten-free diet [34]. However, more recent data indicate that this risk may be lower than previously anticipated [35,36].

### The following extraintestinal symptoms are secondary to malabsorption [2,3]

• Peripheral neuropathy (vitamin B12 and B1 deficiency)

• Anemia (iron, vitamin B12 and folate deficiency)

• Growth failure in children

• Bone pain (osteoporosis and osteopenia, vitamin D and calcium deficiency)

• Muscle cramps (magnesium and calcium deficiency)

• Night blindness (vitamin A deficiency)

• Weight loss (impaired absorption of most nutrients)

• Edema (protein and albumin loss)

• Weakness (hypokalemia and electrolyte depletion)

• Bleeding and hematoma (vitamin K deficiency)

### The following extraintestinal symptoms/manifestations are probably not secondary to malabsorption (atypical CD) [27]

• Neurological disorders such as depression, epilepsy, migraine, ataxia

• Dermatitis herpetiformis

• Elevated liver enzymes, liver failure

• Infertility

• Stomatitis

• IgA nephritis

• Myocarditis

• Idiopathic pulmonary hemosiderosis

• Arthritis

### The following diseases/conditions are associated with celiac disease [29-31]

• Autoimmune diseases such as type 1 diabetes, Sjögren syndrome, thyroid diseases (Hashimoto's thyroiditis and Graves's disease), autoimmune hepatitis and primary biliary cirrhosis

• Selective IgA deficiency

• Turner's syndrome

• Down's syndrome

## Diagnostic methods

Firm diagnosis of CD can only be established after small intestinal biopsies confirming a flat jejunal mucosa with absence of normal intestinal villi [2,3]. Histological examination further demonstrates a cellular infiltrate of lamina propria consisting of plasma cells and lymphocytes [37]. The number of intraepithelial lymphocytes and especially the number of gamma/delta T cells is markedly increased (>40 IEL/100 epithelial cells) [38,39]. Small intestinal changes can vary from a nearly normal mucosa with increased IEL [40] to a completely flat mucosa [41]. Pathologists write a standardized report to characterize the histological features of celiac disease [42] (see table 2). In cases with minor histological changes, a six-week gluten challenge and subsequent endoscopy can be performed. In subjects with negative serology (see below), diseases other than CD that can cause villous flattening must be ruled out [1]. Thus, the diagnosis of celiac disease should not depend only on biopsy, but also the clinical picture and serology should be considered.

**Table 2:** The modified Marsh classification [42].

	**Type 0**	**Type 1**	**Type 2**	**Type 3a**	**Type 3b**	**Type 3c**
IEL	<40	>40	>40	>40	>40	>40
Crypts	Normal	Normal	Hypertrophic	Hypertrophic	Hypertrophic	Hypertrophic
Villi	Normal	Normal	Normal	Mild atrophy	Marked atrophy	Absent

* Numbers are given as intraepithelial lymphocytes (IEL)/100 epithelial cells

Antibody tests cannot replace histology but are very helpful as a screening tool in asymptomatic subjects at higher risk of developing celiac disease (first-degree relatives and patients with autoimmune diseases, *e.g*. diabetes) [43]. Until recently, the determination of IgA endomysium antibodies was the most important laboratory test in the diagnosis of CD [3,44]. In some scientific research institutions this test reaches a sensitivity and specificity around 97%. However, in routine laboratories, this test is much less sensitive (giving rise to more false-negative results) [45,46]. Since frozen sections of monkey esophagus are used for this assay, it is very expensive. The autoantigen, which is recognized by endomysium antibodies (EMA), is now discovered and shown to be tissue transglutaminase (tTG). Subsequently, several ELISA tests for detection of tTG antibodies were developed. They have the same sensitivity and specificity as EMA assays [47-49]. Occasionally, tTG antibodies may detect CD patients undiagnosed by endomysial antibodies and *vice versa *[50]. These new tests are cheaper and the results obtained are much better reproducible. The first generation of tests used guinea pig tTG. They were less sensitive and specific [51] than the new tests that use human transglutaminase [52-54]. However, the quality of the different tTG-test kits can also differ markedly [54]. Consequently, strong false positive tTG results were reported in the clinical practice [55].

For routine diagnosis, the determination of gliadin antibodies are no longer required [56,57]. They are less sensitive and specific than EMA and tTG antibody tests. The sensitivity of IgA gliadin antibodies is around 80–90% and the specificity around 85–95%. IgG gliadin antibodies are even less sensitive (75–85%) and specific (75–90%). However, they allow the detection of patients with both celiac disease and IgA deficiency. Patients with IgA deficiency may have negative IgA-gliadin, EMA and tTG tests. IgA-tTG in combination with IgG-tTG antibody assays (to detect subjects with IgA deficiency) have frequently replaced all other tests [58-63].

Seroconversion of tTG antibodies after the initiation of a gluten-free diet is not necessarily accompanied with morphological recovery of the mucosa. After one year of a gluten-free diet a substantial number of celiac patients turned negative for tissue transglutaminase or endomysial antibodies but still manifested villous atrophy [64,65]. The normalization of the mucosa can take several years [66]. On the other hand, some patients might have positive tissue transglutaminase antibodies but a completely normal mucosa. Thus, after the initiation of a gluten-free diet, antibody tests are not very helpful to draw a conclusion about the condition of the mucosa. The determination of IgA-tTG antibodies might be helpful to monitor the success of the gluten-free diet [61]. However, others reported that their negativity is a falsely secure marker of strict diet compliance [67].

## Management including treatment

Two guidelines about the management of CD were recently published: Recommendations of the North American Society for Pediatric Gastroenterology, Hepatology and Nutrition [56] and National Institutes of Health (NIH) consensus development conference statement on celiac disease [68]. Once the diagnosis of celiac disease has been firmly established (see diagnostic methods), gluten has to be immediately withdrawn. Dietary gluten is present mainly in wheat, rye and barley [2,41,69]. Since small amounts of gluten are hidden in many food products, dietary counseling is absolutely necessary. In most countries, support groups for celiac disease greatly help patients by providing them with adequate dietary information.

Gluten is also found in oats, however many studies suggested that the ingestion of oats is safe for most patients [70-74]. This data was in line with recent studies, which failed to identify the toxic amino acid sequences in oats (see below) [75]. Despite these observations, the ingestion of oats can not be endorsed, since commercial oats are often contaminated with wheat or rye. In addition, the oats-containing gluten-free diet caused in some individuals more intestinal symptoms than the traditional diet. Rarely, mucosal integrity was disturbed and more inflammation was evident in the group on oats diet. Nevertheless, oats may provide an alternative in the gluten-free diet; at the same time, patients should be aware that the intestinal symptoms may worsen [76,77]. Antibodies to oat prolamines were more frequently found higher in children with CD [76]; however, the significance of this finding is not clear.

According to the European Society of Pediatric Gastroenterology and Nutrition (ESPGAN) criteria, repeated endoscopy with jejunal biopsy is not necessary if the patient's condition improves after introducing a gluten-free diet [78]. The results of repeated endoscopy could be rather confusing since normalization of the histology may take up to eight years [66]. At the same time, persistent mucosal abnormalities were described despite a strict gluten-free diet [79,80]. Thus, there is no point in repeating endoscopy when the patient improves on a gluten-free diet. Vitamin supplementation may be necessary at the beginning of a gluten-free diet in subjects with severe celiac disease. Patients with clinically overt CD should go on a strict gluten-free diet since those not treated are at a higher risk of malignancies [34], anemia and osteoporosis [13,17,81]. In addition, the onset of autoimmune diseases which are associated with celiac disease seem to be related to the duration of exposure to gluten [82]. However, this issue is controversial especially in adults: negative reports have been published in Italy and Finland [83,84].

In subjects who do not respond to a gluten-free diet, non compliance or inadvertent ingestion of gluten should be considered [85]. Microscopic colitis in patients with persistent diarrhea should be ruled out by colonoscopy [86]. Small intestinal bacterial overgrowth was reported to be frequently present in CD [87]. Patients with this condition can be identified by a hydrogen breath tests (H2 breath test). They rapidly improve after the initiation of an antibiotic regimen. When these conditions are ruled out, other diseases such as intestinal lymphoma [88] or refractory sprue should be envisaged [89].

Whether asymptomatic screen-detected patients (with normal laboratory values and no gastrointestinal symptoms) should adhere to a gluten-free diet remains a controversial issue [90,91]. Some authors suggest the silent cases of CD to be treated with a life long gluten-free diet, otherwise they are exposed to the risks of long-term complications. On the other hand, 1) until now, no study has proven the benefit of a gluten-free diet for this subgroup of patients; 2) the compliance of these subjects to follow a gluten-free diet is known to be very low; 3) the relative risks for lymphomas and gastrointestinal cancers in patients with CD was found lower than previously thought [35,92,93] with no elevated cancer risk during childhood and adolescence [35,36].

## Etiology

Celiac disease develops in patients who have ingested gluten, which is present in wheat, rye and barley (recent reviews: [2-4]). Gluten proteins are grouped into high molecular weight (HMW) glutenins, low molecular weight (LMW) glutenins and alpha-, gamma- and omega-gliadins (based on their differential N-terminal sequence, electrophoresis size and mobility) [94]. Until recently it was thought that the toxic proteins causing CD are not present in the HMW glutenins. In consequence, development of non-toxic HMW glutenins-containing food products was proposed (*e.g*. transgenic food). However, recent evidence showed that there is no group of gluten proteins safe for patients with CD [94]. Interestingly, gluten extracted from several ancient wheat species was reported incapable to stimulate T cell lines (previously shown to be responsive to toxic gliadin fragments) (see below) [95]. These findings raise the prospect of identifying bred wheat species with low or absent levels of harmful gluten proteins.

Based on the high association between human leukocyte antigens (HLA) and celiac disease (over 95%) [96], it is thought that HLA-DQ2 positive antigen-presenting cells have gliadin peptides toxic to CD4+ T cells. CD4+ T cells drive the immune response and damage the mucosa [97-102]. Tissue transglutaminase (tTG) was identified as the autoantigen, which is recognized by the endomysial antibodies [57,103] (see below).

Tissue transglutaminase, an enzyme essential in wound healing for all individuals, was shown to be able to deamidate gliadin peptides *in vitro *[98-101]. The modified gliadin peptides bind much better to HLA-DQ2 and elicit a stronger T cell response. Furthermore, gliadin is one of the main substrates of tTG. It was reported that several peptides can be cross-linked and that tTG itself can be incorporated into HMW complexes. These newly generated molecules are potential "neo"-autoantigens, which might be responsible for the induction of a destructive immune response.

Gliadin-reactive CD4+ T cells were isolated from the intestinal mucosa of patients with celiac disease [97]. These T cells can elicit a B cell response with production of autoreactive antibodies against gliadin peptides or tTG. However, whether the role of these antibodies is significant [104] or whether they have a pathogenic role in celiac disease remains unknown [105]. It also remains unknown why tTG or gliadin complexes are recognized only by T cells from celiac patients and not by those from healthy HLA-DQ2 positive subjects. In addition, the increase in CD8+ T cells in the epithelium of the mucosa (IEL) cannot be explained by HLA-DQ2 positive cells, which only activate CD4+ T cells. Additional factors like infections, which damage the mucosa may trigger the onset of the disease in predisposed individuals.

In recent years, considerable progress has been made in identifying the amino acid sequences of gliadin peptides that may trigger CD. From these studies it became evident that there is not just one peptide but many gliadin peptides, which are capable to stimulate T cell lines from patients with CD [106-109]. All toxic gliadin fragments had a high content of the amino acid proline [75,106,107]. A "super" gliadin 33 amino acids peptide, capable to stimulate all gliadin specific T cell lines in a very vigorous manner, has been identified by the group of Shan L *et al*. [110]. This gliadin fragment is rich in proline and contains multiple short amino acid sequences, which were previously shown to stimulate T cell lines [106,111-113]. In addition, this peptide was completely resistant to the breakdown of endogenous proteases and peptidases, indicating that the full length fragment can reach the small intestine and stimulate mucosal T cells. The same research group described a bacterial prolyl endopeptidase (from *Flavobacterium meningosepticum*), which is capable to digest *in vitro *all proline rich peptides, including the 33 amino acid long "super" gliadin fragment [114]. The authors suggested that patients with celiac disease might be able to tolerate gluten by supplementing the food with this enzyme. Currently, this hypothesis is under investigation (see Celiac Sprue Research Foundation [115]; Gluten detoxification trial). However, it is unlikely that all toxic gluten peptides would be efficiently destroyed, so this enzyme treatment would fail to prevent the gluten toxicity completely [108,116].

## Genetic counseling

Celiac disease is associated with *HLA-DQ2/DQ8 *in over 95% of the cases [102]. However, 20% of the healthy population carry the same gene and will never develop celiac disease. Thus, no genetic test which could identify "celiac genes" is currently available. Furthermore, there is no need for genetic screening since celiac disease can be treated by a specific diet and patients usually enjoy a good quality of life and a normal life expectancy. In subjects with uncertain diagnosis of CD, the determination of the *HLA *genes might be helpful [117]. *HLA-DQ2/DQ8 *negative subjects are highly unlikely to suffer from CD.

## Unresolved questions

Celiac disease is the only autoimmune disease in which the agents that trigger the disease are identified, *i.e*. gliadin, the autoantigen transglutaminase and even the *HLA*-genes (DQ2/DQ8) which are associated with the disease. However, the exact mechanisms damaging the intestinal mucosa and the exact role of autoantibodies such as tTG and EMA antibodies in the pathogenesis of the disease remains unsolved. Furthermore, the question of how much gluten is toxic (*e.g*. trace amounts of contaminated gluten) is also a matter of discussion in both Europe and USA.
